# Predictive Periodontitis: The Most Promising Salivary Biomarkers for Early Diagnosis of Periodontitis

**DOI:** 10.3390/jcm10071488

**Published:** 2021-04-03

**Authors:** Carlo Cafiero, Gianrico Spagnuolo, Gaetano Marenzi, Ranieri Martuscelli, Michele Colamaio, Stefania Leuci

**Affiliations:** Department of Neurosciences, Reproductive and Odontostomatological Sciences, University of Naples, Federico II, 80131 Naples, Italy; c.cafiero@unina.it (C.C.); gaetano.marenzi@unina.it (G.M.); r.martuscelli@unina.it (R.M.); michele.colamaio@libero.it (M.C.); stefania.leuci@unina.it (S.L.)

**Keywords:** predictive periodontology, lab-on-a-chip, host-derived diagnostic markers, salivary biomarkers, periodontitis

## Abstract

The primary cause of tooth loss in the industrialized world is periodontitis, a bacterial anaerobic infection whose pathogenesis is characterized by composite immune response. At present, the diagnose of periodontitis is made by a complete status check of the patient’s periodontal health; full-mouth plaque score, full-mouth bleeding score, probing depth, clinical attachment level, bleeding on probing, recessions, mobility, and migration are evaluated in order to provides a clear picture of the periodontal conditions of a single patient. Chair-side diagnostic tests based on whole saliva could be routinely used by periodontists for a very early diagnosis of periodontitis, monitoring, prognosis, and management of periodontal patients by biomarker detection, whose diagnostic validity is related to sensitivity and specificity. Recent paper reviews and meta-analyses have focused on five promising host derived biomarkers as candidate for early diagnosis of periodontitis: MMP-8 (Metalloproteinase-8), MIP-1α (Macrophage inflammatory protein-1 alpha), IL-1 β (Interleukin-1 beta), IL-6 (Interleukin-6), and HB (Hemoglobin), and their combinations. Chair-side Lab-on-a-chip (LOC) technology may soon become an important part of efforts to detect such biomarkers in saliva medium to improve worldwide periodontal health in developed nations as well as in underserved communities and poor countries.** Their applications in preventive and predictive medicine is now fundamental, and is aimed at the early detection of risk factors or the presence or evolution of the disease, and in personalized medicine, which aims to identify tailor-made treatments for individual patients. The aim of the present paper is to be informative about host derived periodontal biomarkers and, in particular, we intend to report information about the most important immune response derived biomarkers and Hemoglobin as candidates to be routinely utilized in order to obtain a chair-side early diagnosis of periodontal disease.

## 1. Introduction

An amazing evolution in dental research has been recorded in the last few years, revealing the intimate mechanisms at the base of Periodontitis, a genetically linked pathology determined by Gram negative anaerobic infection and characterized by composite immune reactions as a response to bacterial load. At the present time, periodontal diagnosis is made by performing a complete clinical status check of the patient’s periodontal health (full-mouth plaque score, full-mouth bleeding score, probing depth, clinical attachment level, bleeding on probing, recessions, mobility, migration) supported by digital photographs and periapical X-rays. At present, the periodontal defense strategy is almost totally reactive because periodontists only make a start when periodontal infection has already begun, determining the damage of periodontal tissues [1].

**Figure 1:**
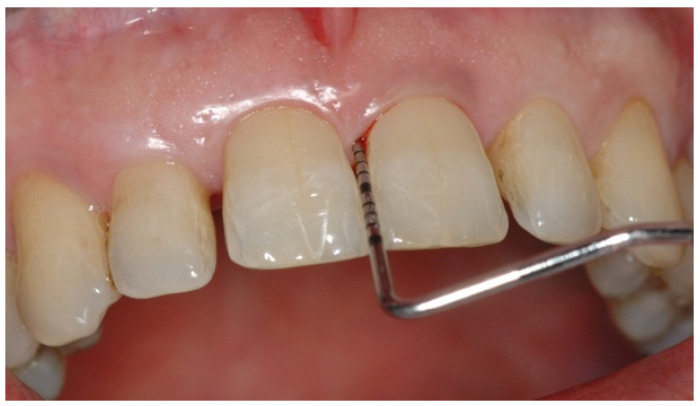
Bleeding on Probing (BoP): Blood coming out during probing is at this time the unique predictive test used by periodontists.

The unique predictive test used by periodontists for routinely checking the stability or progression of periodontitis is “Bleeding on Probing, (BoP)” [2], recorded by inserting a periodonatal probe at the bottom of the gingival sulcus or periodontal pocket (Figure 1).

Blood coming out from the bottom of the pocket can be recorded during probing: BoP that is repeatedly positive (BoP+) is a predictor of future loss of attachment (activity phase) in 30% of cases (positive predictive value); meanwhile, BoP repeatedly negative (BoP−) is a predictor of periodontal health in 98% of cases (negative predictive value) [3,4,5]. In addition, a functional diagram to evaluate the patient’s risk of recurrence of periodontitis (“Spider’s web”) has been proposed [6]. At this time, the instruments that periodontist have to make diagnosis of periodontitis are mainly related to a “periodontal reactive approach” because, at this time, we can use very few “periodontal predictive“ instruments.

The purpose of a “futuristic” periodontal diagnosis in the near future will be to diagnose periodontal disease before it comes clinically detectable in order to early stop its progression by the use of biomarkers. Biomarkers are biological indicators with high prognostic and predictive value that can be related to the onset or development of a pathology. They must be capable of being measured accurately and quickly and must have a high prognostic or predictive value. In short, they must be able to predict the presence of a disease or its progression, if it is a disease marker, or to give indications on the most appropriate type of drug and response, if it is a treatment response marker. Their applications in preventive and predictive medicine is now fundamental, and is aimed at the early detection of risk factors or the presence or evolution of the disease, and they can also be applied in personalized medicine, which aims to identify tailor-made treatments for individual patients. The aim of the present paper is to be informative to clinicians who are not familiar with the details of periodontal biomarkers; in particular, we intend to report information about the most important host derived biomarkers and among them the immune response derived biomarkers and Hemoglobin as candidates that can be routinely utilized in order to obtain a chair-side early diagnosis of periodontal disease. The most important therapeutic topic is that, by the use of periodontal biomarkers, we could “intercept” periodontal disease at a very early stage, when periodontal tissues lesions are not yet clinically detectable and, as a consequence, we can treat the disease early in order to stop it before periodontal lesions are established.

## 2. Immune Response in Periodontitis

Worldwide periodontists have to make a huge cultural effort in changing the actual “reactive” therapeutic point of view into a futuristic “predictive” one. On account of this, it appears very important (i) to identify a periodontal initial lesion when it is not yet clinically detectable and (ii) to intercept the so called “active phase” of periodontitis. In order to get this result, clinicians need hi-tech diagnostic tools in order to detect the specific biomarkers that are released during the early phases of immune response. The host-microbial equilibrium constitutes the situation for clinically healthy periodontal tissue; when plaque bacterial load occurs it determines an important immune response, which releases many substances in periodontal tissues, and some of them could be eligibly as biomarkers for the early diagnosis of Periodontitis.

The most important cells involved in pathogenesis of periodontal diseases are Polymorphonuclear Leukocytes (PMN), Macrophages (Mø), and Osteoclasts [1]. Their functions in periodontal immune response are briefly described below.

In this brief description of the pathogenesis of periodontitis, we focused on the immunological mechanisms and related cells leading to the releasing of molecules eligible as biomarker candidates for an early diagnosis of periodontitis.

### 2.1. Polymorphonuclear Leukocytes (PMN) Activation

PMN leukocytes, representing the first line of defense of periodontal tissues, can cause tissue damage as a result of their accumulation in gingival epithelial tissues. Further tissue damage can be caused by a variety of enzymes and oxygen metabolites that are released by PMN during the immune reaction. The result of these activities is that the junctional epithelium becomes filled with ulcers, allowing the passage of bacteria underneath connective tissue.

Neutrophil collagenase, also known as matrix metalloproteinase-8 (MMP-8) is one of the most representative enzyme involved in the breakdown of the extracellular matrix in Periodontitis. The primary function of MMP-8 is the degradation of type I, II, and III collagens, determining periodontal attachment loss.

### 2.2. Macrophage (Mø) Activation

The second line of defense is mostly represented by macrophages. They play a decisive role in controlling bacterial diffusion in the connective tissue and represent an important source of enzymes, cytokines, and inflammatory mediators such as Interleukin-1 β (IL-1 β), Tumor Necrosis Factor-α (TNF-α), Prostaglandin E2 (PGE2), Transforming Growth Factor-β (TGF-β), and Macrophage inflammatory protein-1 alpha (MIP-1α/CCL3). The primary functions of these molecules are reported below:

- IL-1 β is released by LPS-activated macrophages, lymphocytes, and fibroblasts. It stimulates Mø and fibroblasts to secrete PGE2 and causes osteoclastic differentiation and activation [7];
- TNF-α is principally secreted by LPS-stimulated macrophages and lymphocytes and causes osteoclastic differentiation and activation [8];
- PGE2 causes vasodilatation, vasopermeability, and resorption of the alveolar bone;
- IL-1 β, TNF-α and PGE2 stimulate fibroblasts and Mø to release Metalloproteinases (MMPs), urokinase plasminogen activator (u-PA), tissue inhibitor of metalloproteinases, PGE2, TGF-β, and interleukin-1 receptor antagonist [9];
- MIP-1α belongs to the family of chemotactic cytokines [10]. It is secreted by macrophages and performs several functions, such as recruiting inflammatory cells, wound healing, inhibition of stem cells, and activation of bone resorption cells, and it directly induces bone destruction. Cells that secrete MIP-1α are increased at sites of inflammation and bone resorption. MIP-1α plays an important role in the pathogenesis of various inflammatory diseases and conditions that exhibit bone resorption, such as periodontitis. Biological fluids from patients with these diseases exhibit elevated levels of MIP-1α [11].

### 2.3. Osteoclast Activation

**Figure 2:**
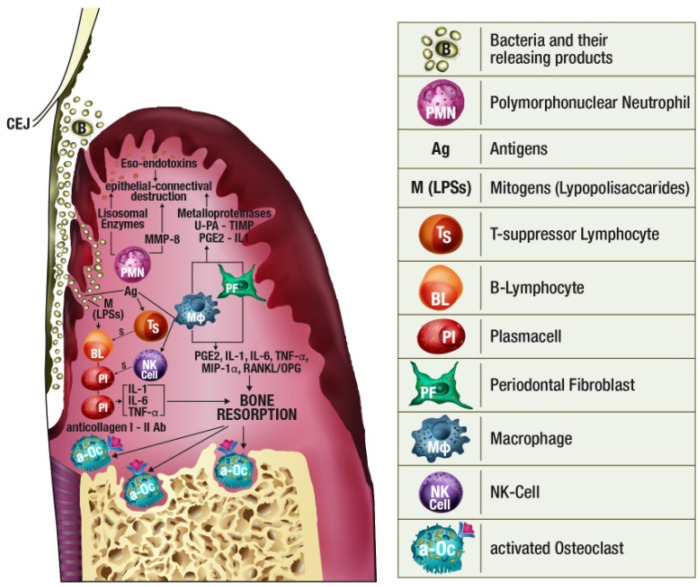
Polymorphonuclear (PMN) leukocytes, representing the first line of defense of periodontal tissues. They release several products during the immune reaction. MMP-8 determines degradation of type I, II, and III collagens.

Many substances (PGE2, IL-1, IL-6, TNF-α) secreted by Mø, fibroblasts, plasma cells, and T lymphocytes are involved in osteoclastic activation. The receptor activator of NF-kB ligand (RANKL) promotes osteoclastic differentiation and the inhibition of osteoclast apoptosis. Under physiological conditions, RANKL produced by osteoblasts binds to RANK on the surface of osteoclast precursors. RANKL is up-regulated by Parathyroid hormone (PTH), and IL-1. Osteoprotegerin (OPG) is produced by fibroblasts and constitute a false target for RANK, inhibiting, as a consequence, the osteoclastic activation [12,13] (Figure 2).

## 3. Immune Response Host Delivered Salivary Products as Biomarkers for Early Periodontal Diagnosis

The word “biomarker” refers to substances in biologic samples that may predict a disease state in a single patient. Nevertheless, the word has evolved to include genomic or proteomic analyses that could also predict “a response to a drug (efficacy, toxicity, or pharmacokinetics) or indicate an underlying physiologic mechanism” [14]. 

Many dental associations, such as the American Dental Association (ADA), recognize the importance of scientific research on oral fluid diagnostics [15]. In the near future, the use of a chair-side lab-on-a-chip (LOC) to detect biomarkers for several dental disorders will be desirable in routine dentistry. Industry and research should walk side by side to provide to operators, in a short time, LOC in order to diagnose periodontal disease and other oral diseases at early stages, dealing with extremely small whole saliva volumes in order to detect biomarkers. Oral fluid (whole saliva) includes glandular-duct saliva and gingival crevicular fluid in which many substances derived by the immune response may be detected [16]. Recently, several authors have affirmed that host delivered biomarkers could play a crucial role in the early diagnosis of periodontitis [17,18]. In the last few years, several systematic reviews and meta-analyses have focused on the analysis of host derived biomarkers detected in saliva in order to identify among them the most eligible ones for diagnosis of periodontitis [19,20]. The diagnostic validity of a single biomarker is related to its sensitivity and specificity. “Sensitivity is the ability to detect a disease in patients in whom the disease is truly present (i.e., a true positive), and specificity is the ability to rule out the disease in patients in whom the disease is truly absent (i.e., a true negative)” [14]. 

Over the last decade, the entire human salivary proteome has been reported on, revealing that 1166 proteins are present in human saliva [21,22], and several have been focused as biomarkers for periodontal diseases [23]. 

Among them, four clusters of markers released during the immune response may be eligible as biomarkers for periodontitis: (1) host-derived enzymes; (2) tissue breakdown products; (3) host response modifiers [24]; (4) Cytokines.

### 3.1. Host-Derived Enzyme

These are released during the immune response, principally by immunocompetent cells elicited by bacterial load.

#### 3.1.1. Alkaline Phosphatase

This is produced by neutrophils, fibroblasts, osteoblasts, osteoclasts, and several bacteria. Its amount appears higher in the active sites than in the inactive ones in the course of periodontitis. Its elevated level in Gingival Crevicular Fluid seems to express early attachment loss [25].

#### 3.1.2. Beta-Glucuronidase

This is a lysosomal enzyme whose amount appears higher in active vs. inactive ones [25]. Lamster et al. showed a predictive value of a high level of Beta-glucuronidase in relation to clinical attachment loss [26].

#### 3.1.3. Cathepsin B

Cathepsin B is mainly released by macrophages in activity sites; meanwhile, it appears reduced after periodontal treatment [27,28,29,30].

#### 3.1.4. Metalloproteinase-8 (MMP-8, Collagenase-2)

MMP-8 appears to be the most promising host derived enzyme as a biomarker for the progression of periodontitis vs. stable periodontitis [31,32].

#### 3.1.5. Metalloproteinase-9 (MMP-9, Gelatinase)

MMP-9 appears elevated in patients with recurrent attachment loss. Its levels decrease significantly following periodontal therapy [33].

#### 3.1.6. Dipeptidyl Peptidases II and IV

These are principally secreted by neutrophils, lymphocytes, macrophages, and fibroblasts. 

Their main function lies in the degradation of periodontal collagen tissue. In sites with attachment loss, very high levels of both enzymes were reported [34].

#### 3.1.7. Metalloproteinase-12 (MMP-12, Elastase)

This is released from the first line of immune defense, which are neutrophils and macrophages. Higher elastase levels are demonstrated in active sites compared to inactive ones [35,36,37].

### 3.2. Tissue Breakdown Products

These are released subsequent to the destruction of periodontal tissues, determined directly by bacteria toxins/enzymes, or as a collateral effect of the immune response following bacteria invasion.

#### 3.2.1. Pyridinoline Cross-Linked Carboxyterminal Telopeptide of Type I Collagen (1-CTP)

This represents a molecule derived from collagen tissue degradation, whose detection in gingival crevicular fluid is a biomarker of periodontal disease [38].

#### 3.2.2. Chondroitin-4-Sulphate (C-4-S)

This is a bone-specific glycosaminoglycan detected in untreated chronic periodontitis sites; a statistically significant correlation between the gingival crevicular fluid (GCF) level of C-4-S, PPD, and CAL has been reported [39].

#### 3.2.3. Hemoglobin (HB)

Hemoglobin is a protein localized in red blood cells transporting oxygen to tissues and carbon dioxide from tissues to lungs. Hb can be revealed in Gingival Crevicular Fluid using salivary occult blood tests (SOBTs). SOBTs have been evaluated as a screening method for periodontal status in order to discriminate subjects with a poor periodontal status [40,41,42,43,44,45,46,47,48,49,50,51].

### 3.3. Host Response Modifiers

Receptor activaton of the nuclear factor-Kb (RANK)/Osteoprotegerin (OPG)/Receptor activator of nuclear factor-Kb ligand (RANKL) system can be detected in the gingival tissue and whole saliva. 

RANK—Expressed by osteoclasts and their precursors—Activated by RANK Ligand binding.

OPG protein is secreted by osteoblasts/bone lining cells—Natural inhibitor of RANK Ligand—Blocks RANK Ligand signaling to balance bone remodeling. 

RANKL is secreted by osteoblasts, fibroblasts, bone marrow stromal cells, and activated T and B cells [52].

RANKL binds to RANK on the surface of preosteoclasts, activating them in osteoclasts. RANKL is up-regulated by OPG. RANKL is increased whereas OPG is decreased in periodontitis compared to healthy gingiva or gingivitis. 

When OPG binds to RANKL, the signal between marrow stromal cells and osteoclast precursors is inhibited; this situation determines a decrease of osteoclastogenesis (Figure 1) [53,54,55]. The balanced regulation of the RANKL-osteoprotegerin expression system can determine health from disease, as demonstrated in a number of bone destructive diseases, including bacterial arthritis, rheumatoid arthritis [56], periodontitis [57], and, lately, peri-implantitis [58,59].

### 3.4. Cytokines

#### 3.4.1. Macrophage Inflammatory Protein-1α (MIP-1α)

MIP-1α is a chemotactic cytokine (chemokine). Monocytes, macrophages, activated eosinophils, and fibroblasts are the sources of these proteins [10,60]. The main effect of MIP-1α mainly consists of chemotaxis and transendothelial migration, affecting monocytes, T lymphocytes, dendritic cells, NK cells, and platelets [60].

#### 3.4.2. Interleukin-1 β (IL-1 β)

IL-1 β is a pro-inflamatory cytokine expressed particularly by mononuclear phagocytic lineage such as macrophage, NK cells, monocytes, and neutrophils [61,62], but it is also produced by endothelial cells, keratinocytes, synovial cells, osteoblasts, glial cells, and numerous other cells. The main effects of Il-1 β are (i) Endothelial cells activation, (ii) Neutrophils diapedesis induction, and (iii) Enhancement of lymphocytes (T and B) cytokines synthesis [63,64]. One of the most important biologic activities of IL-1 is as a lymphocyte activating factor. IL-1 enhances the production of IL-2 T lymphocyte–derived, which determines the increase of B-cellproliferation and in consequent increasing immuno-globulin synthesis. Interaction of IL-1 with the central nervous system is responsible for producing fever. Moreover, IL-1 stimulates bone resorption and collagen deposition [65].

#### 3.4.3. Interleukin-6 (IL-6)

This is produced by osteoblastic cells, gingival fibroblasts, gingival lymphocytes, and macrophages stimulated by IL1. It appears as a fundamental factor in the regulation of bone remodeling because it acts by increasing bone resorption determined by osteoclasts activated by IL1 [66].

#### 3.4.4. Tumour Necrosis Factor-α (TNF-α)

TNFα stimulates the proliferation and differentiation of osteoclasts precursors and also acts on mature osteoclasts, activating them [67,68].

#### 3.4.5. Tumour Necrosis Factor-β or Lymphotoxin (TNF-β or LT)

This performs many biological activities similar to those of TNF-α, stimulating bone resorption; moreover, it has a negative effect on bone formation, as it inhibits both collagen synthesis and non-collagenic protein synthesis by osteoblasts [67,68,69].

#### 3.4.6. Interferon-γ (INF-γ)

IFN-γ is produced by natural killer (NK) cells as a part of the innate immune response, and by CD4 (Th1, T helper cells) and CD8 cells (Tc, cytotoxic T lymphocyte) as part of specific immune response [70]. IFN-γ is also produced by non-cytotoxic innate lymphoid cells (ILC), a family of immune cells first discovered in the early 2010s [71,72]. INF-γ is a central factor in the regulation of bone resorption because it can function as a pro- or antiresorptive cytokine [72,73,74,75], but the reason why IFN-γ has variable effects in bone is unknown.

## 4. The Most Promising Host Derived Biomarkers as Candidates for Early Diagnosis of Periodontitis and Their Combination

At present, well-studied molecules collected in oral fluid (whole saliva) associated with host response factors have been proposed as diagnostic biomarkers for periodontitis [16].

Over 65 components detected in oral fluid have been examined as possible markers for the progression of periodontitis (for a complete review, see [76]).

Among them, five promising biomarkers have been identified as eligible candidates for the diagnosis of periodontitis, and their combination has been evaluated in order to enhance sensibility and specificity of the molecular analysis.

### 4.1. Most Promise Biomarkers

Recent systematic reviews and meta-analyses [19,20,77] have identified five promising host derived biomarkers as good candidates to be elected for the early diagnosis of periodontitis:

- **Metalloproteinase-8 (MMP8):** An enzyme released by PMN during immune reaction [78]. Salivary and systemic levels of MMP8 appear to be valuable biomarkers for both acute coronary syndrome (ACS) and periodontitis [79,80]. Recent reports have shown that local and systemic levels of aMMP-8 can reflect the grading and staging of periodontitis [81,82]. In terms of sensitivity, Arias-Bujanda N et al. [19] showed a value of 72.5%, according to de Lima et al. [83]. Other authors have reported MMP-8 as one of the strongest markers for tissue destruction, with sensitivity ranging from 65% to 87%, and specificity ranged from 48% to 87% [84,85];
- **Macrophage inflammatory protein-1 alpha (MIP-1α):** Secreted by macrophages increased at the sites of periodontal inflammation and bone resorption [86]. Its increased level can reveal the hidden presence of subclinical inflammation in periodontal clinically healthy sites [87], and it can also discriminate periodontitis in type II diabetics (T2DM) patients. Non-surgical periodontal treatment can affect the salivary level of MIP-1α [88]. It appears associated with periodontal bone remodeling, showing high sensitivity and specificity of 95% and 97%, respectively [89];
- **Interleukin-1beta (IL-1β)**: Released by LPS-activated macrophages (Mø), lymphocytes, and fibroblasts. It stimulates Mø and fibroblasts to secrete PGE2, determining bone destruction [90] and fibroblasts, and Mø releases Metalloproteinases (MMPs), determining connective tissue destruction. Genetic variations of IL-1β + 3954 appear to be associated with increased risk of periodontitis in Koreans (Detection of association between periodontitis and polymorphisms of IL-1beta + 3954 and TNF-alpha −863 in the Korean population after controlling for confounding risk factors) [91]. For IL-1β, the sensitivity ranged from 54% to 88% and specificity ranged from 52% to 100% across five studies [31,84,85,92,93]. Clinical parameters showing periodontitis such as gingival index (GI), probing depth (PD), and GCF flow were significantly correlated with gingival crevicular fluid (GCF) and tissue IL-1beta activity [94];

**Interleukin-6 (IL-6):** A pro-inflammatory cytokine secreted by macrophages in response to specific bacteria and by osteoblaststs to stimulate osteoclastic activity.

- The levels of salivary IL-6 appear to be increased in patients affected by Chronic Periodontitis as compared to healthy controls [95]. Interleukin-6 572C/G and RS1800796 polymorphisms appear as genetic risk factors for periodontitis patients in the Asian population [96,97]. Its sensitivity ranged from 52% to 80%, and specificity ranged from 48% to 87% [31,84,85,93];
- **Hemoglobin(HB):** This has a sensitivity value of 72% and a specificity value of 75% [19]. SOBTs may offer a simple screening method for periodontal status when clinical periodontal examination is not possible, although this test it is not sufficiently specific to be a suitable surrogate for a periodontal clinical examination [48]. Mäkinen et al. [49] reported the presence of hemoglobin (Hb), detected in the GCF of periodontal disease sites. In addition, Hanioka et al. [50] observed the existence of Hb in the GCF of mild periodontal pockets. They speculated that invisible bleeding has previously occurred in a pocket with early periodontitis in spite of the negative finding by BOP inspection (BOP–). This hypothesis was supported by other studies, which suggested that the detection of Hb derived from microbleeding in gingival sulci may serve as an index for preclinical diagnosis [51,98] (Table 1).

Recently, other proteins have been proposed as promising biomarker of periodontitis:

- **Salivary neuropeptides (vasoactive intestinal peptide, VIP and neuropeptide Y NPY)** showed significantly higher levels in the saliva of patients with periodontitis and were correlated with bleeding on probing scores in patients with periodontitis [99];
- **Oxidative stress-related biomarkers (OS)** in saliva and gingival crevicular fluid associated with chronic periodontitis has been reported in a systematic review and meta-analysis. A direct link between CP and OS-related bio- marker levels in the local site has been suggested by a significant decrease of total antioxidant capacity and a significant increase of malondialdehyde (MDA), nitric oxide, total oxidant status (TOS), and 8-hydroxy-de-oxyguanosine levels in the saliva of CP patients [100];
- **MicroRNAs (MiRNA-146a and miRNA155)** provide consistent, non-invasive, diagnostic and prognostic biomarkers that can be used to monitor periodontal health status in saliva among diabetic and non-diabetic patients [101];
- **Salivary oxidative stress biomarkers and advanced glycation end products** were investigated in a cross-sectional study in patients affected by periodontitis and in periodontally healthy patients with type 2 diabetes and corresponding systemically healthy controls. Salivary 8-hydroxy-2’-deoxyguanosine (8-OHdG) alone, or in combination with 4-hydroxy-2-nonenal (4-HNE), advanced glycation end products (AGE) and AGE receptor (RAGE) for diabetics, and salivary 8-OHdG alone, or in combination with malondialdehyde (MDA) and high sensitivity C-reactive protein (hsCRP) for systemically healthy persons, could potentially serve as non-invasive screening marker(s) of periodontitis [102];
- **Soluble Neuropilin-1 (sNRP-1)** is a glycoprotein with angiogenic and immune regulatory functions positively related to periodontitis and could probably be involved in the pro-inflammatory mechanisms observed in periodontal clinical tissue inflammation [103].

### 4.2. Combination of Biomarkers for Earliest Diagnosis of Periodontitis

Many investigators are interested in combining biomarkers to forecast a binary outcome or detect underlying disease [104]. The combination of some of the previously described biomarkers appear to show a very high sensitivity and specificity in order to diagnose periodontitis.

Distinction between gingivitis and periodontitis groups has been analyzed by only one study, which reported sensitivity of 81% and specificity of 71% for the combination of IL-6 and MIP-1α; meanwhile, a combination of IL-1β, IL-6, MMP-8, and MIP-1α was found to have a good sensitivity of 78% and specificity of 78% [84].

The combination of IL-6 and MMP-8 showed, for periodontitis vs. healthy gingiva, a high sensitivity of 94% and a specificity of 100% [85].

Diagnostic precision was at the maximum for the combination of IL-1β, IL-6, and MMP-8, with sensitivity and specificity range of 78–94% and 77–97%, respectively [84,85].

An outstanding predictive value of 98% was reported for paired combinatory analysis of IL-1β and MMP-8 and IL-1β and IL-6, as well as the triple combination of IL-6, MMP-8, and IL-1β. Finally, an ideal positive predictive value of 100 was calculated for the combination of IL-6 and MMP-8 [85].

## 5. Conclusions

Oral fluid is the mirror of periodontal health. Unfortunately, its importance in the diagnosis of periodontitis as well as of other oral diseases is still underestimated. We think that it is important to underline that, today, dentistry does not appear to be in line with the times in terms of managing biomarkers. The current deficit in the development of new diagnostic strategies is a cultural deficit for which a global strategy is needed in order to favor a modern cultural approach to the diagnosis of oral diseases. In order to fill the cultural gap, it is necessary to update the scientific knowledge of dental operators through a global cultural strategy aimed to involve:

(i) Universities, in teaching predictive dentistry (e.g., the creation of a specific subject in degree courses in dentistry, post-graduate courses, and PhD courses);
(ii) Dental researchers, in the publication of scientific papers on biomarkers as diagnostic tools for oral diseases;
(iii) Industries, which should provide chair-side LOC to be used by dental operators.

**Figure 3:**
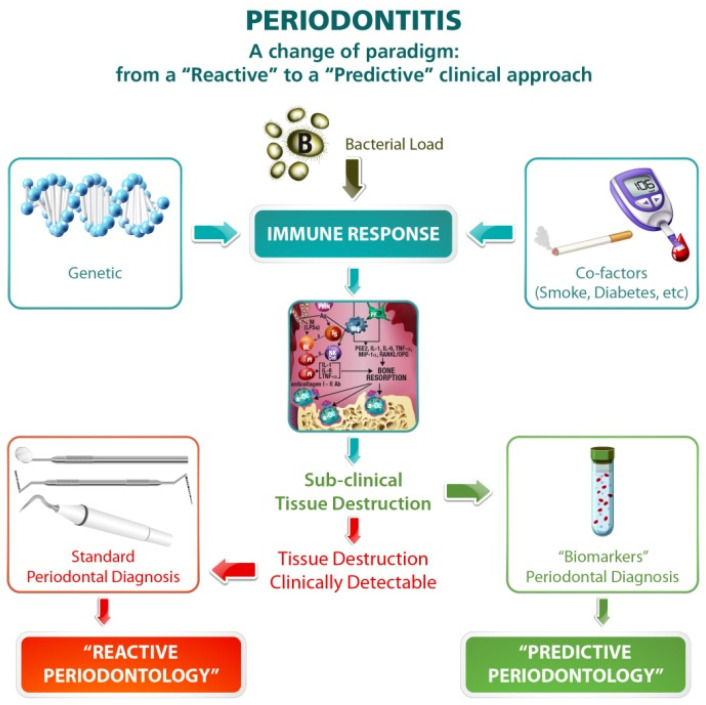
A change of paradigm in periodontal diagnosis is desirable. A shift from a “Reactive“ approach, in which clinicians react to the presence of clinically evident periodontal damage, towards a “Predictive” approach, in which the disease is intercepted early when it is already in a sub-clinical phase, is the new objective in periodontal diagnosis.

Advances in microfluidics technology are revolutionizing molecular biology procedures for enzymatic analysis, DNA analysis, and proteomics [105]. Digital microfluidics appears promising for future application to diagnose periodontal diseases by the use of a chair-side Lab-on-a-chip technology able to detect the periodontal biomarkers released during the immune response [106,107,108]. We have to go a long way if we are to change a traditional “reactive” approach to a “predictive” one (Figure 3), but the pathway has already been outlined; it is “time for new guidelines in advanced healthcare” in dentistry [109].

## Figures and Tables

**Table 1:** Early diagnosis of periodontitis: Sensitivity and Specificity of the most promising host derived biomarkers.

Releasing Cells	Biomarker	Sensitivity %	Specificity %
Polymorphonuclear Leukocytes	MMP8 (Metalloproteinase-8)	72% [19,79] 65.87% [80,81]	48–87% [80,81]
Macrophage	MIP-1α (Macrophage inflammatory protein-1 alpha):	95% [82]	97% [82]
Macrophage Lymphocytes Fibroblasts	IL-1 β (Interleukin-1 β)	54–88% [31,80,81,83,84].	52–100% [31,80,81,83,84]
Macrophages Osteoblaststs	IL-6 (Interleukin-6)	52–80% [31,80,81,84]	48–87% [31,80,81,84]
Red Cells	Hemoglobin (HB)	72% [19]	75% [19]
